# Treatment of soft tissue radionecrosis with intact fish skin graft

**DOI:** 10.1016/j.jdcr.2025.05.002

**Published:** 2025-05-28

**Authors:** Elina V. Zhivov, Morgan Vague, Alex G. Ortega-Loayza

**Affiliations:** a Dr. Phillip Frost Department of Dermatology and Cutaneous Surgery, University of Miami, Miami, Florida; b Department of Dermatology, Oregon Health and Science University, Portland, Oregon

**Keywords:** intact fish skin graft, skin graft, soft tissue radionecrosis, ulcers, wound healing

## Introduction

Soft tissue radionecrosis (STRN) is a delayed complication of radiation therapy (XRT), characterized by impaired blood supply to irradiated areas, resulting in chronic tissue breakdown and impaired wound healing.^1^ Approximately 50% of patients diagnosed with malignancy are projected to receive XRT as part of their treatment.^2^ While XRT effectively reduces the burden of various cancers, 85% to 95% of patients experience dermal tissue injury following therapy,^3^ with 3% to 5% developing STRN—a painful and refractory condition characterized by nonhealing ulcers that markedly impact quality of life.^4^^,^^5^

Conventional therapies, such as topical corticosteroids and specialized wound dressings, are often ineffective in managing STRN. More advanced approaches, such as hyperbaric oxygen therapy (HBOT), which enhances angiogenesis through elevated oxygen exposure and is covered by Medicare services,^6^ yield inconsistent results. Specifically, a systematic review of HBOT for STRN revealed predominantly grades III to V evidence studies, with highly variable success rates, ranging from 33% to 85%.^7^ Moreover, many of the studies included surgical interventions, suggesting that HBOT is often used as an adjunctive therapy rather than a standalone treatment. This underscores the variable nature of HBOT’s contribution to STRN ulcer healing and highlights the challenge in assessing its true effectiveness without accounting for concurrent treatments.

Here, we present a patient with STRN who failed standard of care^8^ and HBOT, and subsequently was successfully treated with intact fish skin graft (IFSG).

## Case

**Fig 1:**
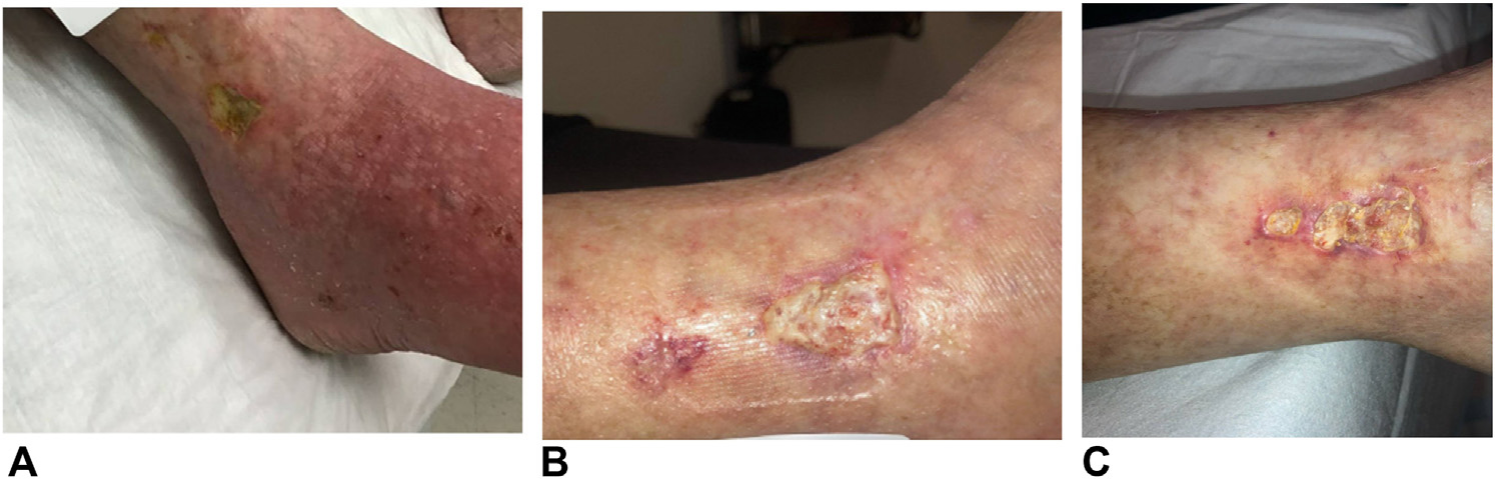
Progression of RLE ulcer. **A,** Ulcer prior to all treatments. **B,** Ulcer prior to HBOT. **C,** Ulcer at the conclusion of HBOT. *HBOT*, Hyperbaric oxygen therapy; *RLE*, right lower extremity.

An 82-year-old female with a history of recurrent squamous cell carcinoma (SCC) of the right lower extremity (RLE) presented with a painful, nonhealing ulcer at the site of her prior SCC. Her SCC was previously managed with Mohs surgery (with secondary intention healing), 3 sessions of electrodessication and curettage, and 20 fractions of low-dose XRT. The ulcer developed within 1 month following completion of XRT. The patient was referred to a wound care clinic and was initially diagnosed with a venous ulcer (Fig 1). Upon presentation, a venous ultrasound of the RLE with triphasic Doppler demonstrated no evidence of reflux or thrombosis. An ultrasound ankle-brachial index was also performed and showed no evidence of peripheral arterial occlusive disease. Subsequently, the patient began weekly sharp debridement and was treated over 8 weeks with topical clobetasol 0.05%, compression therapy (Molnlycke tubigrip and socks), and a range of dressings, including some with antimicrobial properties. The patient endorsed severe pain and required local anesthesia with injectable 4% prilocaine (1 mL) with every debridement. Despite treatment, her ulcer grew from 3.04 cm^2^ to 4.42 cm^2^ and pain intensity remained severe. Shave skin biopsy was performed and showed dermal fibrosis with irregular blood vessels and a lymphohistiocytic mixed infiltrate without evidence of malignancy. Further clinical reassessment showed no evidence of infection or macrovascular compromise, supporting the diagnosis of STRN.^1^^,^^9^ The patient then underwent 60 sessions of HBOT over a span of 12 weeks. While there was improvement in tissue granulation, the ulcer area increased to 6.3 cm^2^ (Fig 1) which prompted referral to dermatology for possible pyoderma gangrenosum.

**Fig 2:**
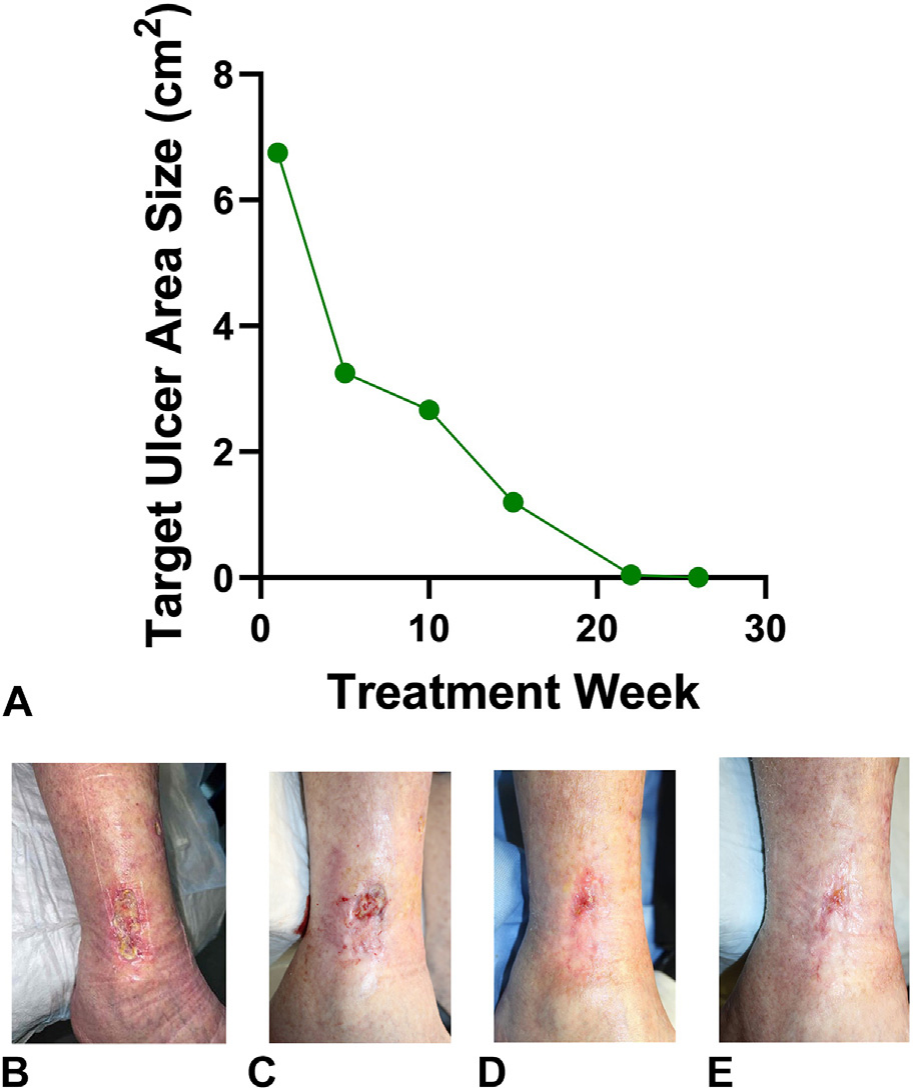
Target ulcer area reduction over time with photographic documentation. **A,** Ulcer area reduction per treatment week. **B,** Ulcer at baseline (week 0, prior to IFSG application). **C,** Ulcer at week 10. **D,** Ulcer at week 22 (final treatment). **E,** Ulcer at week 26 (follow-up). *IFSG*, Intact fish skin graft.

Given the patient’s history of XRT to the affected area, a skin biopsy negative for malignancy, normal venous circulation based on ultrasound, and a PARACELSUS score <10—a validated diagnostic tool for pyoderma gangrenosum that assigns weighted points to clinical and histological features^10^—the diagnosis of STRN was reaffirmed as a diagnosis of exclusion. The patient reported an average pain of 4/10 in the past week and a peak pain of 6/10 in the past 24 hours despite taking 1 to 2 tablets of daily hydrocodone/acetaminophen (5 mg/325 mg). She initially received 8 weeks of standard of care therapy, including sharp debridement, collagen dressing (Promogen Prisma), compression, and was recommended leg elevation. However, the ulcer area remained unchanged, prompting a trial of weekly applications of IFSG (Kerecis MariGen, derived from Atlantic cod, Iceland) combined with continued sharp debridement as needed. Her RLE wound was debrided to expose fresh tissue surface and subsequently irrigated with sterile saline. IFSG was then applied directly to the wound, with the graft carefully molded to fit the wound bed and avoid overlapping the wound edges. Finally, IFSG was secured to the wound using a nonadherent dressing (3M Adaptic). The patient was provided abdominal gauze pads and sterile gauze (Kerlix) in case of wound draining as they were allergic to absorbent foam dressing (Mepilex) and instructed to keep the dressing in place until their follow-up visit 1 week later. After 4 weeks, the patient reported a significant pain reduction on a numeric rating scale. After 22 applications over 6 months, the ulcer completely healed and remained closed at both the 4-week and 12-week follow-up visits (Fig 2). The patient’s clinical course was complicated by cellulitis after 9 IFSG applications, diagnosed by increased erythema and pain with corresponding positive cultures for *Staphylococcus aureus.* It resolved after treatment with oral clindamycin 300 mg TID for 7 days. Infection may have occurred due to the occlusive nature of the dressing.

## Discussion

STRN is an ulcerative condition with no Food and Drug Administration–approved treatments. Current therapy options include HBOT, which improves oxygen supply and accelerates healing, and herbal treatments such as calendula, catechins (epigallocatechin gallate), and aloe vera, which offer anti-inflammatory and antioxidant benefits. Additional options include topical biologics (eg, epidermal growth factor, granulocyte macrophage colony stimulating factor), corticosteroids, and silver-based dressings, which help reduce inflammation, promote tissue repair, and prevent infection. Operative measures are considered a last resort option and are individualized based on patient’s wound characteristics, comorbidities, and physician’s expertise.^11^ The evidence supporting these therapies remains limited, with heterogenous efficacy outcomes.

**Fig 3:**
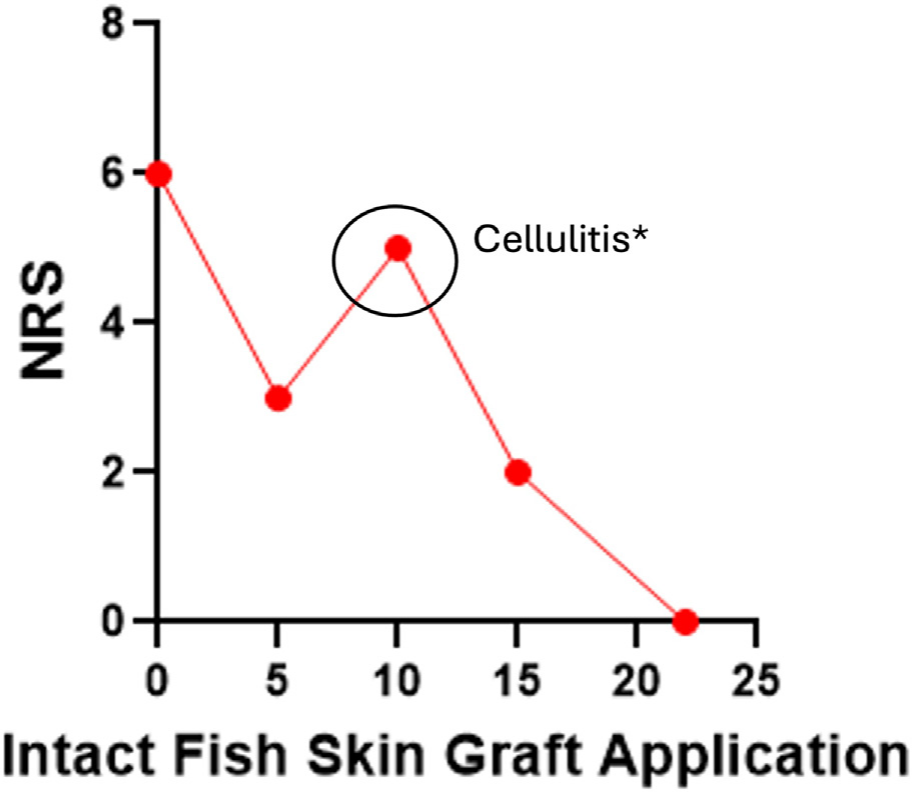
Peak pain level within 24 hours with each intact fish skin graft application. NRS patient-reported pain numeric rating scale. ∗Patient was found to have cellulitis at that visit, which corresponded with an increase in pain. *NRS*, Numeric rating scale.

As therapeutic alternatives have inconsistent outcomes, the use of IFSG in the treatment of STRN is a novel and promising alternative for this challenging to treat skin ulcers. Notably, in addition to promoting wound healing, pain significantly reduced after 4 applications, and halved by 8, allowing us to taper off opioid pain medications. Following 16 applications, the pain was nearly eliminated, with patient’s pain score fluctuating between 0 and 1 until healing (Fig 3).

As STRN arises from compromised blood supply and delayed tissue ischemia, IFSG’s ability to stimulate angiogenesis and promote dermal cells and capillary growth appears to be crucial in enhancing STRN ulcer healing.^12^ Moreover, the healing occurred using IFSG and debridement as needed, without additional interventions. Emerging data support the use of acellular skin matrices as potential treatments for recalcitrant wounds, due to their significant tissue loss and the risk for secondary infection.^12^, ^13^, ^14^, ^15^

In addition to its promising therapeutic effects, IFSG is cost-comparable to other therapies and reimbursed by Medicare, Veterans Administration hospitals, and many insurers.

Given the limited and often unsuccessful therapy modalities available for STRN, clinical trials are warranted to evaluate its efficacy and cost-effectiveness compared to current treatments.

## Conflicts of interest

Alex G. Ortega-Loayza reports consultancy/advisory boards disease-relevant honoraria from Genentech, Boehringer-Ingelheim, Bristol Mayer-Squibb, and Janssen. Alex G. Ortega-Loayza also reports research grants from Lilly, Pfizer, and Janssen. Alex G. Ortega-Loayza is also supported by NIAMS NIH R01AR083110. Elina V. Zhivov and Morgan Vague have no conflicts of interest to declare.
